# Supplementary material for: Leveraging data from the Genomes-to-Fields Initiative to investigate genotype-by-environment interactions in maize in North America
Source: Nat Commun. 2023 Oct 30;14:6904. doi: 10.1038/s41467-023-42687-4 (PMC10616096; doi:10.1038/s41467-023-42687-4)
Supplement: Supplementary file 1 — Supplementary Information [file 41467_2023_42687_MOESM1_ESM.docx]

**Leveraging data from the Genomes-to-Fields Initiative to investigate**

**genotype-by-environment interactions in maize in North America**

Lopez-Cruz *et al.*


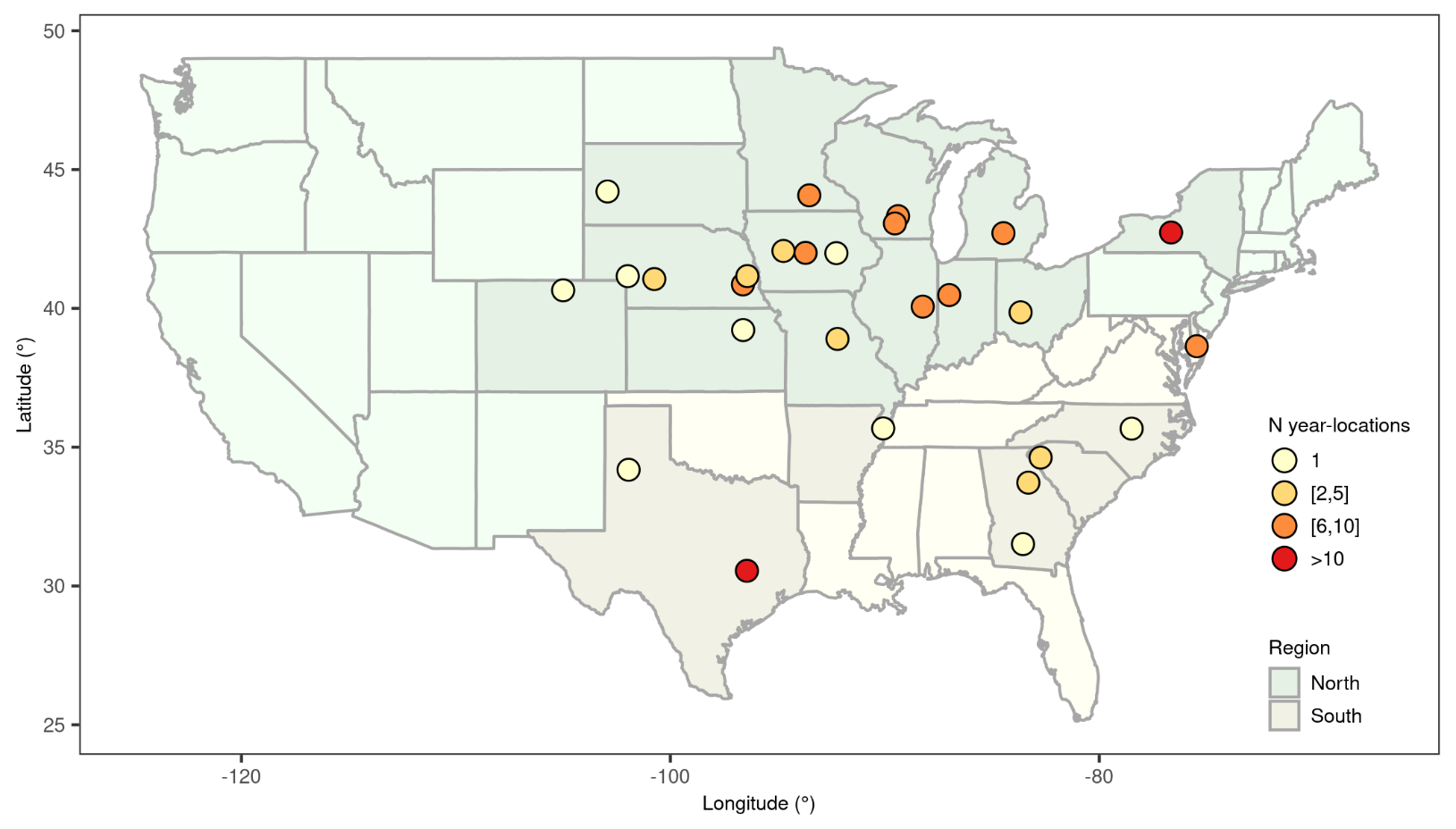


**Supplementary Figure 1. Testing locations in Genomes to Fields trials.** Only non-irrigated locations were used in this study. Map data was obtained from the U.S. Census Bureau (<https://www.census.gov>) which is available through the ‘maps’ R-package^1^ (v.3.40). Source data are provided as a Source Data file.


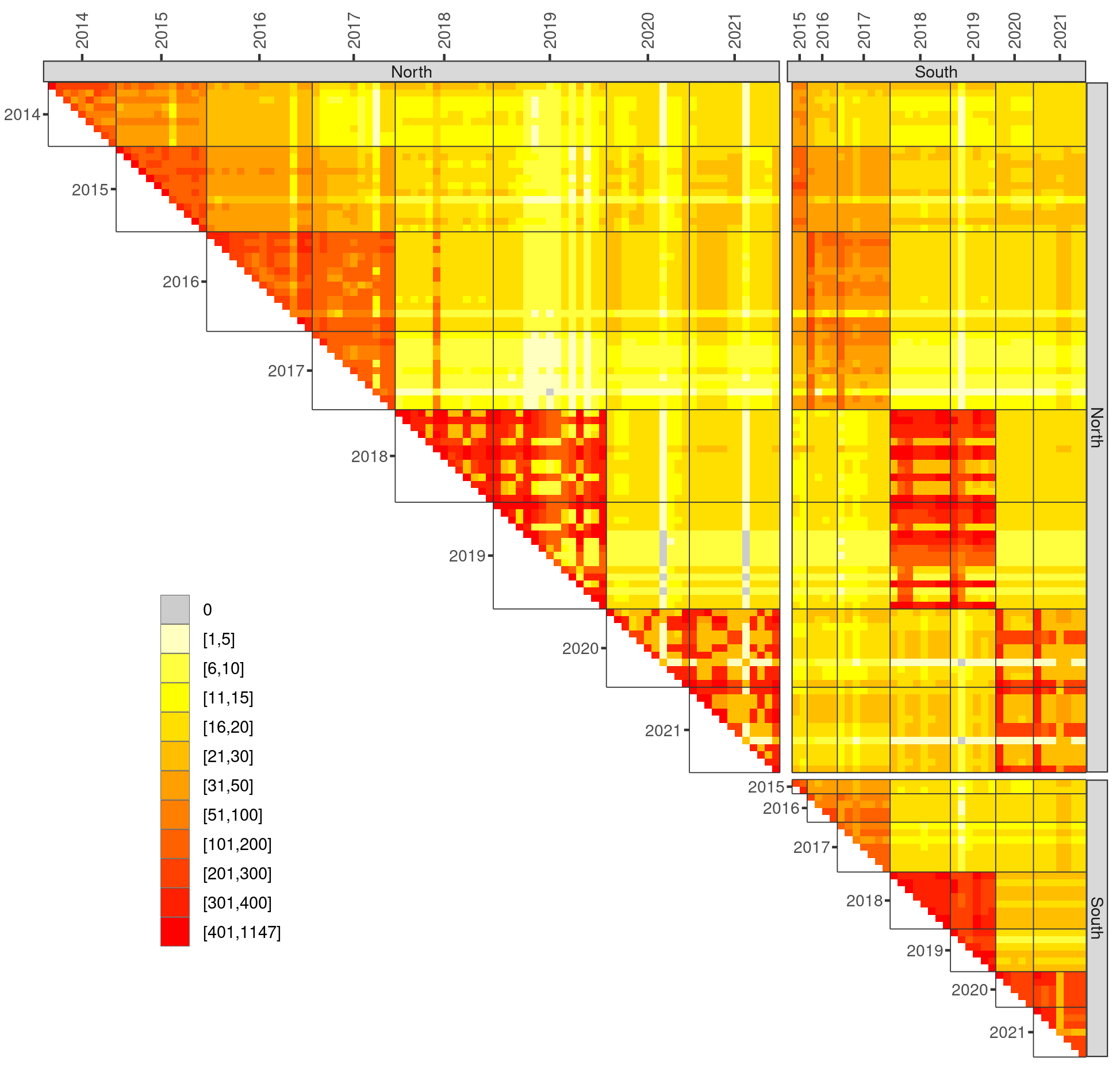


**Supplementary Figure 2. Heatmap showing the number of hybrids in common between year-locations.** The data set includes 136 year-locations (97 in the north region and 39 in the south). Values on the diagonal correspond to the number of unique hybrids evaluated at each year-location. Source data are provided as a Source Data file.


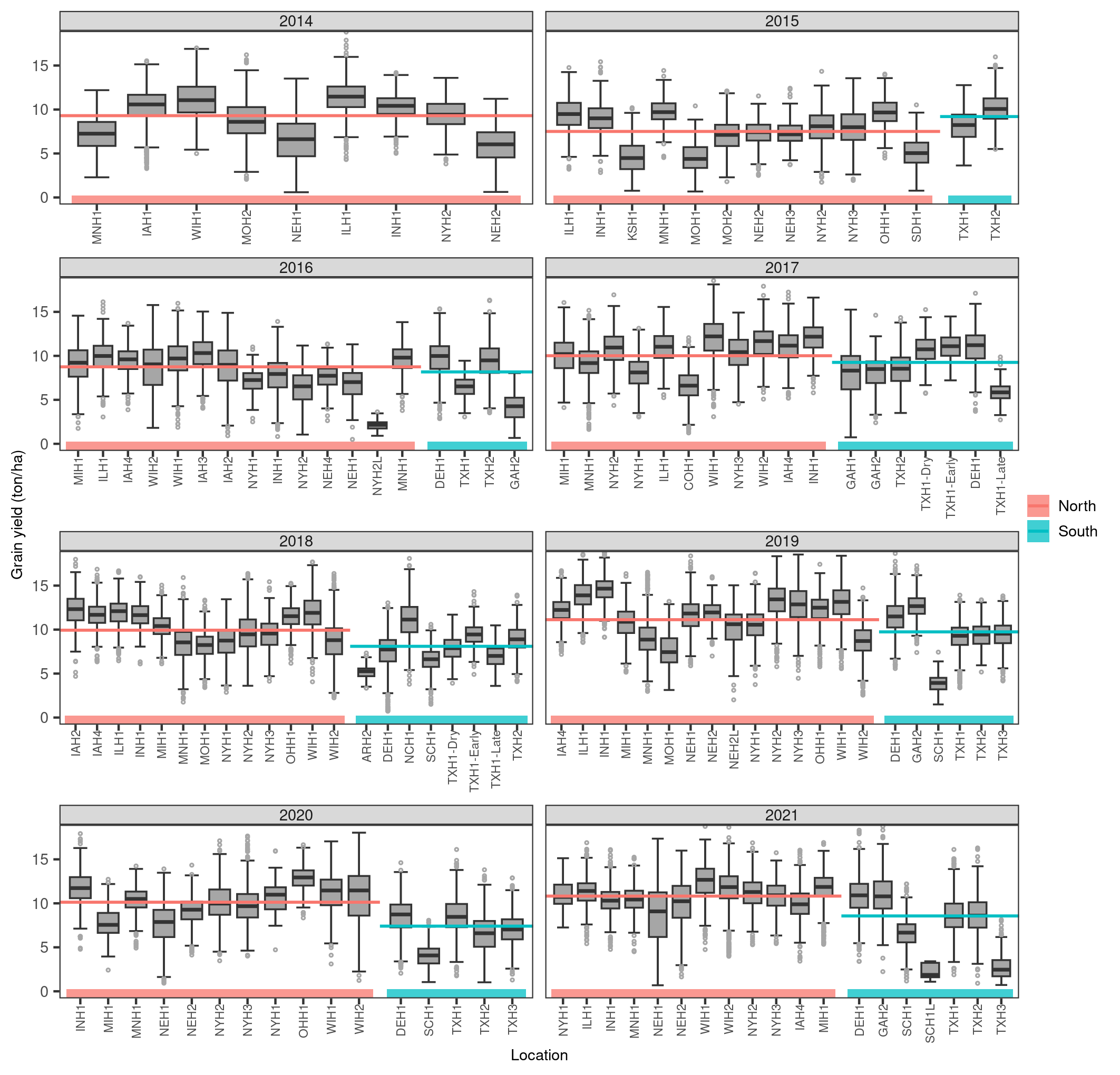


**Supplementary Figure 3. Boxplot of grain yield by year and location within region.** Horizontal lines correspond to the average value across all locations within region. The boxes represent the inter-quartile range (IQR) bounded by the 25^th^ and the 75^th^ percentiles. Line at the center of each box is the median. The whiskers extend from the IQR bounds to $\pm1.5$ times the IQR. Points represent records lying outside the whiskers ends. Source data are provided as a Source Data file.


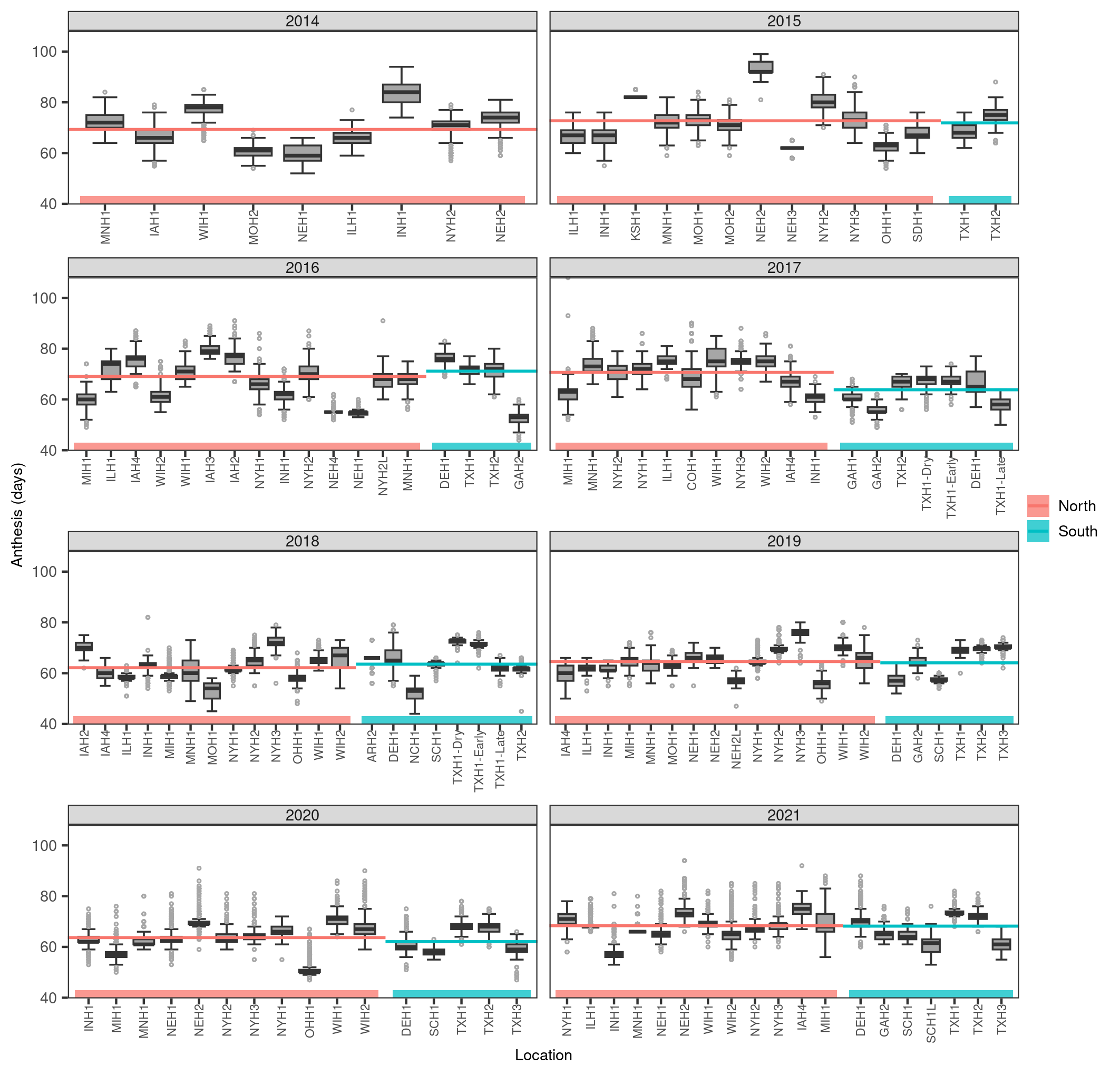


**Supplementary Figure 4. Boxplot of days-to-anthesis by year and location within region.** Horizontal lines correspond to the average value across all locations within region. The boxes represent the inter-quartile range (IQR) bounded by the 25^th^ and the 75^th^ percentiles. Line at the center of each box is the median. The whiskers extend from the IQR bounds to $\pm1.5$ times the IQR. Points represent records lying outside the whiskers ends. Source data are provided as a Source Data file.


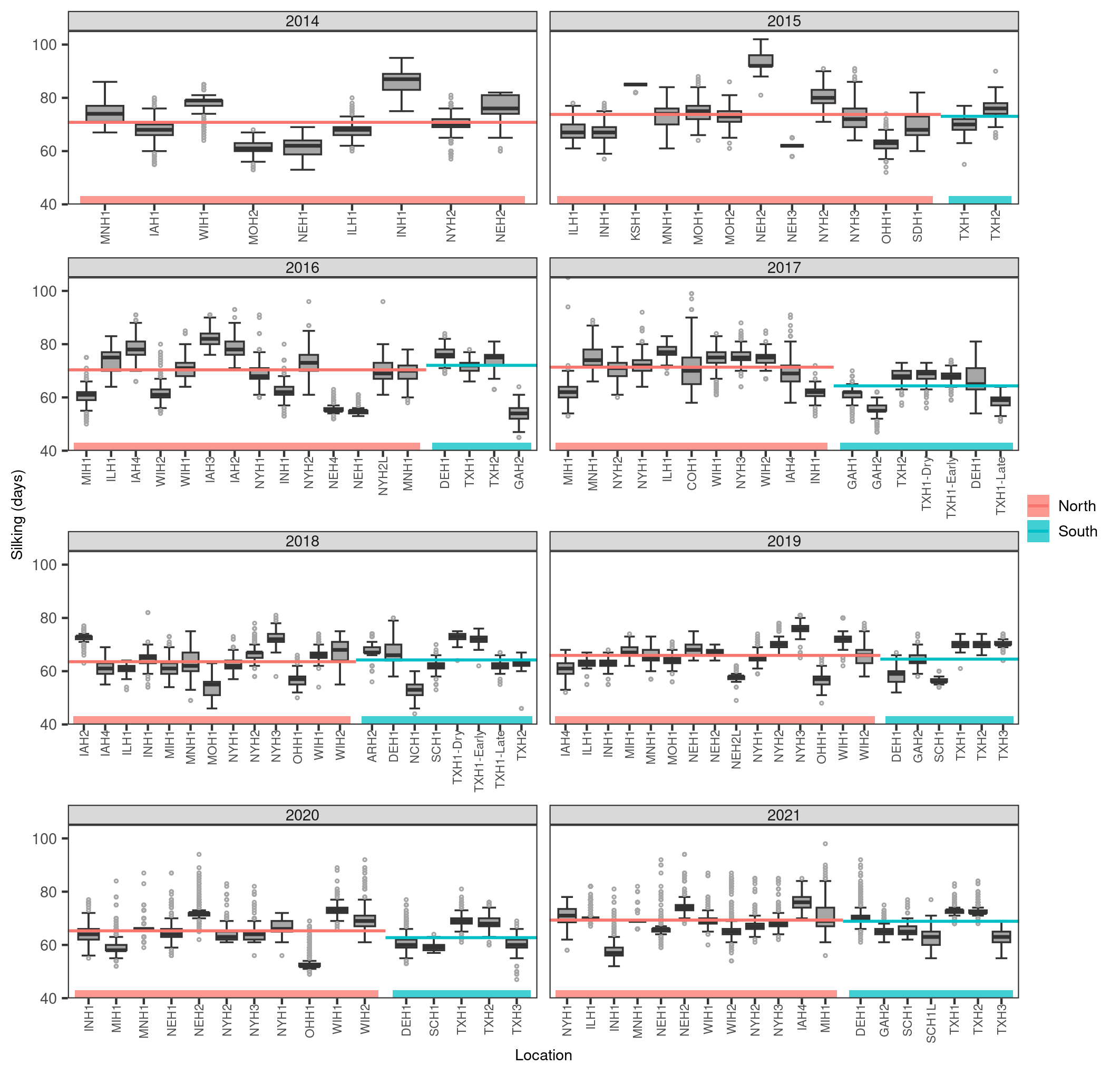


**Supplementary Figure 5. Boxplot of days-to-silking by year and location within region.** Horizontal lines correspond to the average value across all locations within region. The boxes represent the inter-quartile range (IQR) bounded by the 25^th^ and the 75^th^ percentiles. Line at the center of each box is the median. The whiskers extend from the IQR bounds to $\pm1.5$ times the IQR. Points represent records lying outside the whiskers ends. Source data are provided as a Source Data file.


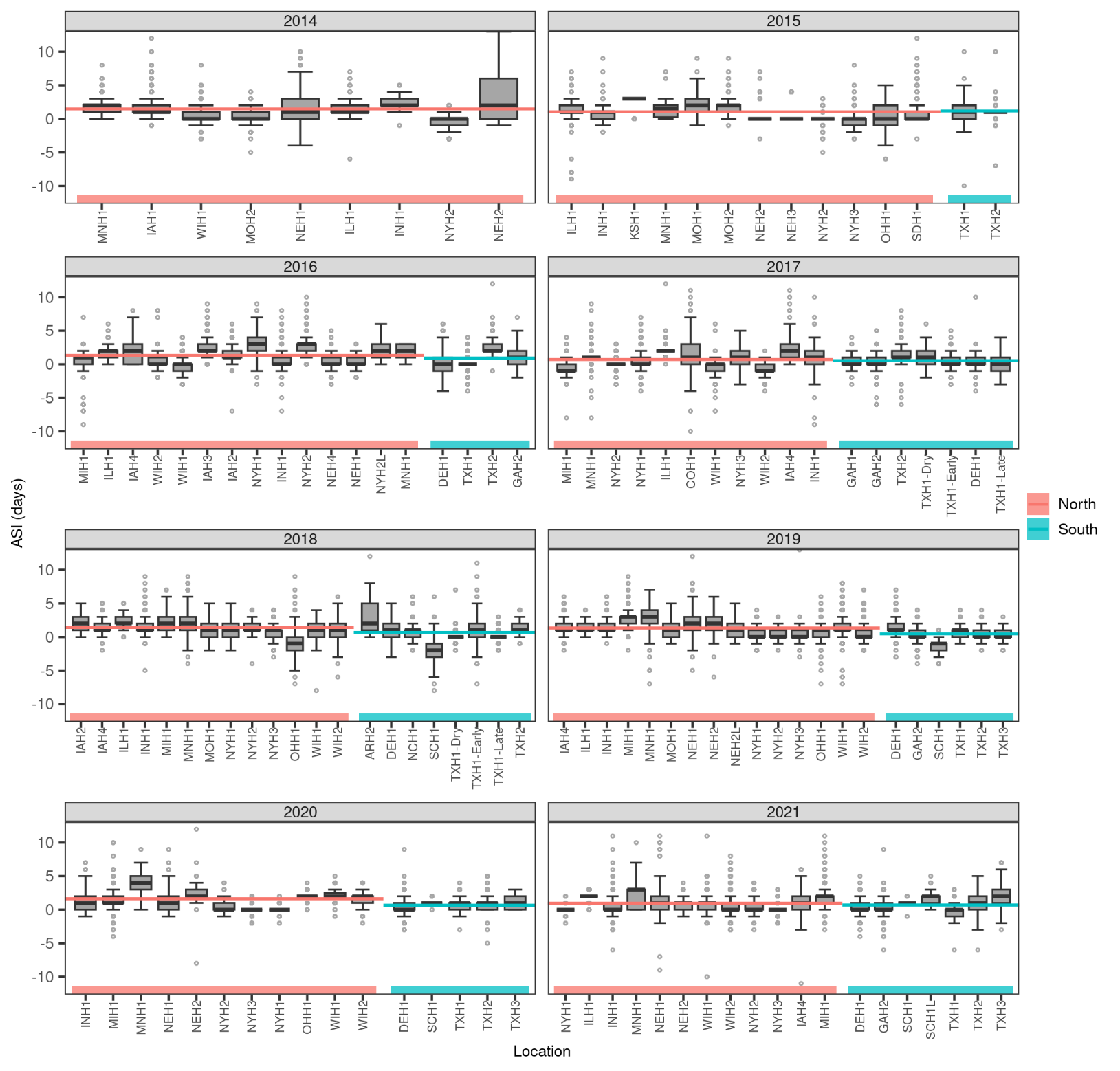


**Supplementary Figure 6. Boxplot of anthesis-silking interval (ASI) by year and location within region.** Horizontal lines correspond to the average value across all locations within region. The boxes represent the inter-quartile range (IQR) bounded by the 25^th^ and the 75^th^ percentiles. Line at the center of each box is the median. The whiskers extend from the IQR bounds to $\pm1.5$ times the IQR. Points represent records lying outside the whiskers ends. Source data are provided as a Source Data file.


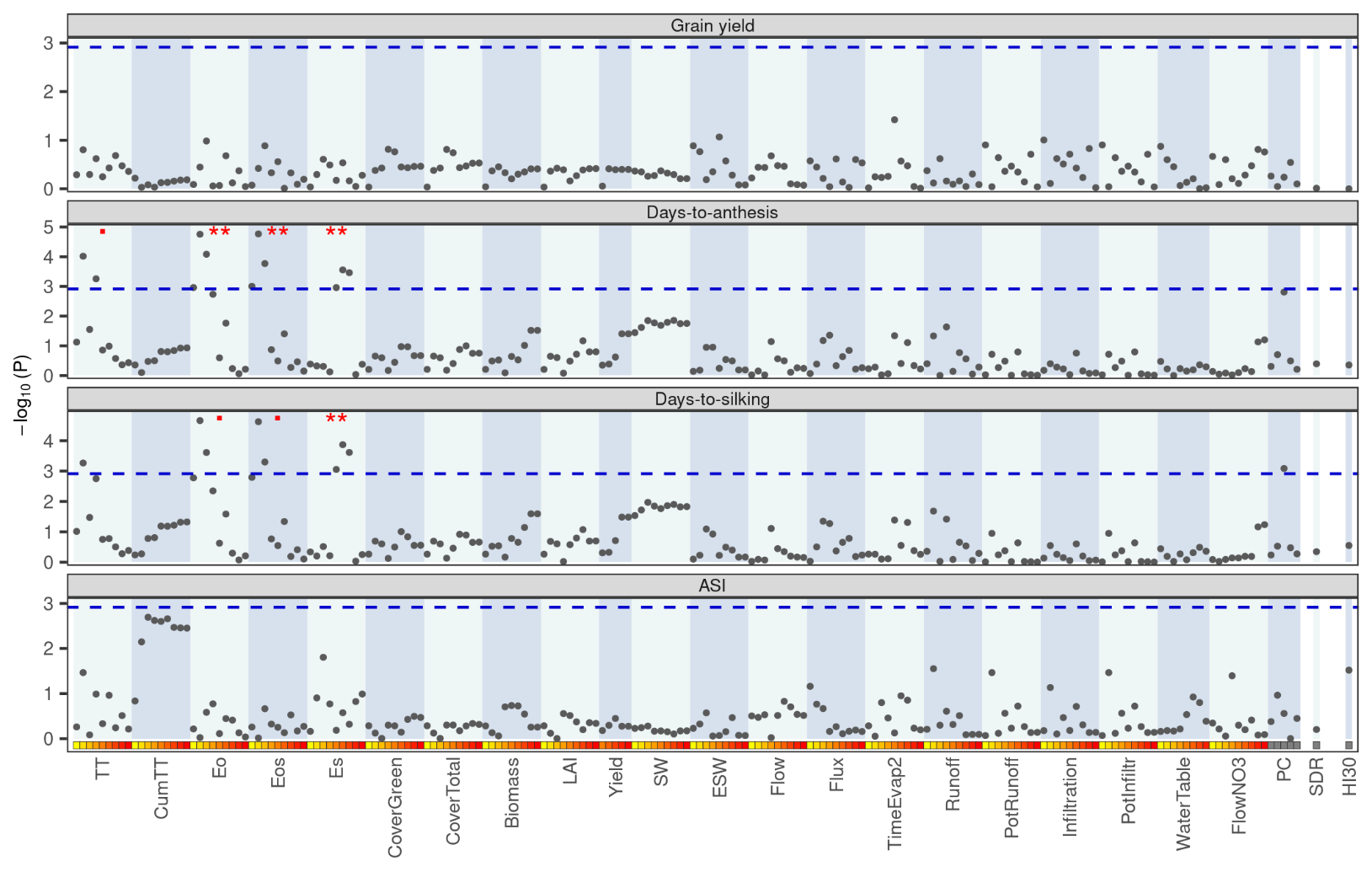


**Supplementary Figure 7.** **Association between individual environmental (co)variates (EC) with yield and flowering traits in the southern trials.** $n=19,617$ records. Each point corresponds to an EC. Association P-values (y-axis) are obtained from the likelihood ratio test (random effects model vs random effects model + EC) using a one-sided chi-square test with 1 degree of freedom. The dashed horizontal line gives the threshold for a 5% significance after Bonferroni adjustment ($P_{adj}=0.0012$). The red marks on the top indicate whether the EC group (i.e., the covariates in the shaded vertical area) was enriched for significant associations in a one-sided Hypergeometric test ($\cdot P\leq0.1$, *$P\leq0.05$, **$P\leq0.01$, and ***$P\leq0.001$). This test was performed only for groups containing more than one covariate. TT=Thermal time, CumTT=Cumulated thermal time, Eo=Potential evapotranspiration, Eos=Potential evaporation, Es=Realized evaporation, LAI=Leaf Area Index, SW=Soil Water, ESW=Extractable soil water, Flow=Unsaturated water movement between layers, Flux=Saturated water flux from each layer to layer below, TimeEvap2=Time since the start of second stage evaporation, PotRunoff=Potential runoff, PotInfiltr=Potential infiltration, FlowNo3=Amount of Nitrogen leaching as NO_3_ from each layer, SDR=Supply-Demand ratio, HI30=Number of days with maximum temperature over 30 °C, and PC=Top 5 PCs derived from all ECs. Source data are provided as a Source Data file.


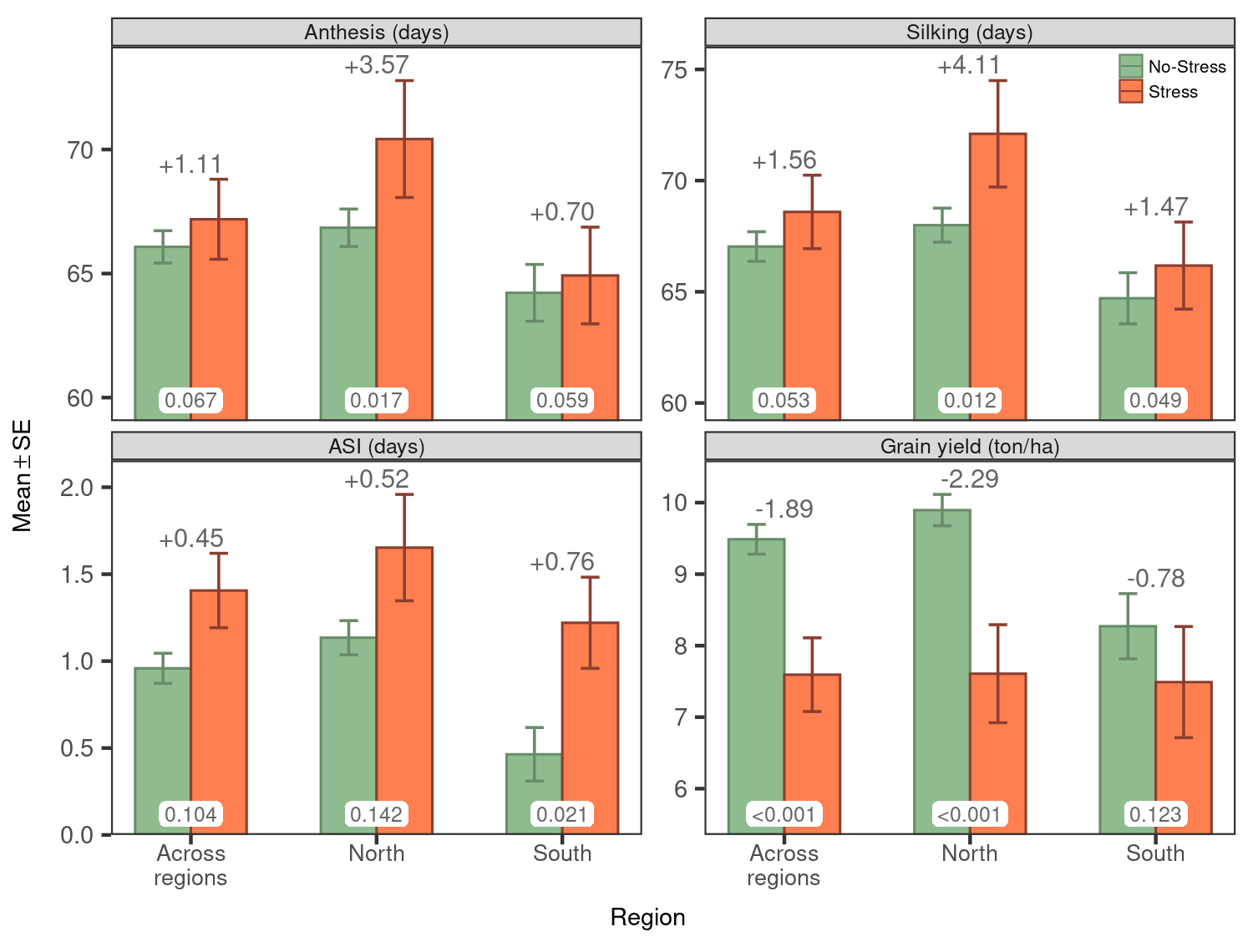


**Supplementary Figure 8.** **Mean (**$\boldsymbol{\pm}$**SE) phenotype difference between year-locations with and without** **combined (drought and heat) stress**. Drought stress (water supply-demand-ratio, SDR$\leq$0.54), heat stress (number of days with temperature over 30 °C, HI30$>$36). $n=59,069$ records (north), $n=19,617$ (south), $n=78,686$ (across regions). Paired bars represent the contrasts intercept (left) and intercept + dummy slope (right) of the random effects model + dummy, where the dummy variable is a fixed effect for SDR$\leq$0.54 & HI30$>$36. Error bars are the standard error (SE) of the contrasts. Numbers on top of bars show the mean difference between stress and no-stress given by the dummy slope. Bottom labels are the P-values of the likelihood ratio test (random effects model vs random effects model + dummy) using a one-sided chi-square test with 1 degree of freedom. Source data are provided as a Source Data file.


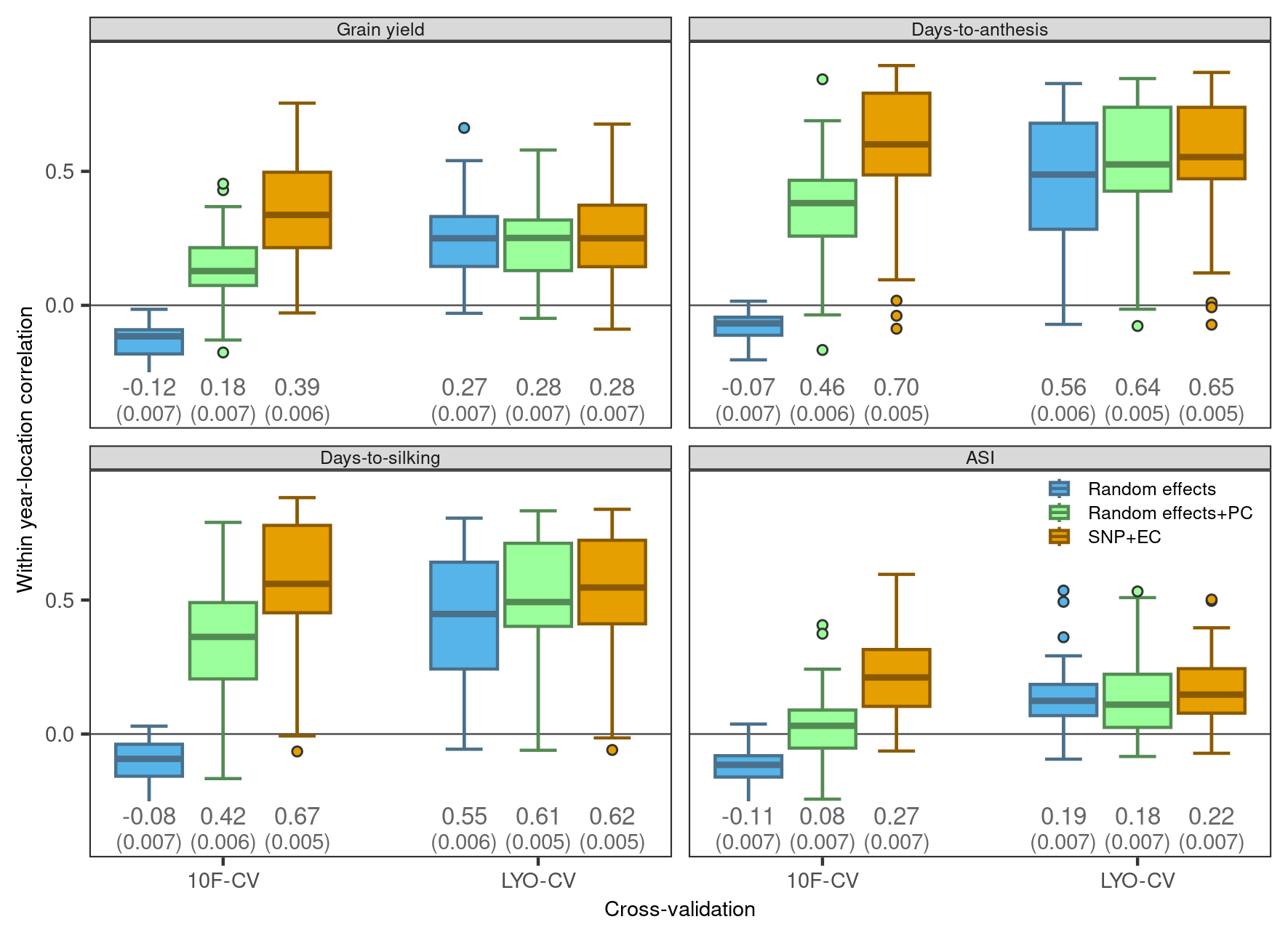


**Supplementary Figure 9. Within year-location correlation between predicted and observed phenotypes for each model and cross-validation (CV) scheme in southern locations**. $n_{YL}=39$ year-locations. The SNP+EC model includes SNPs and environmental covariates (EC). The random effects+PC model includes the top 10 SNPs-derived principal components (PC) plus the top 5 ECs-derived PCs. The boxes represent the inter-quartile range (IQR) bounded by the 25^th^ and the 75^th^ percentiles. Line at the center of each box is the median. The whiskers extend from the IQR bounds to $\pm1.5$ times the IQR. Points represent correlations lying outside the whiskers ends. Numbers at the bottom are the weighted average correlation and standard error (SE, in parenthesis, Eq. **8**). Source data are provided as a Source Data file.

**Supplementary Table 1**. **Testers used to produce the G2F hybrids in years from 2014 to 2021.**

| **Tester**  **group** | **N testers** | **Testers** | **N ***  **hybrids** |
| --- | --- | --- | --- |
| **North** | | | |
| CG | **11** | C103,CG60,CG102,CG110,CG111,CGR01,CGR03,CG44,CG108,CG123,CG120 | 176 |
| DK3IIH6 | 1 | DK3IIH6 | 142 |
| LH | **5** | LH162,LH145,LH38,LH51,LH210 | 50 |
| LH82 | 1 | LH82 | 199 |
| LH198 | 1 | LH198 | 207 |
| LH185 | 1 | LH185 | 223 |
| LH195 | 1 | LH195 | 757 |
| PH | **23** | PHN82,PB80,PHG35,PHM49,PHR25,PHW03,PHHV4,PHRE1,PHG29, PHG47,PHG83,PHR55,PHW30,PHJ65,PHN11,PHN47,PHR03,PHW53, PHM57,PHR63,PHAJ0,PH207,PHW65 | 261 |
| PHB | 1 | PHB47 | 255 |
| PHK | **3** | PHK76,PHK05,PHK56 | 398 |
| PHP | **2** | PHP02,PHP60 | 385 |
| PHT | 2 | PHT69,PHTD5 | 572 |
| PHZ | 1 | PHZ51 | 659 |
| TX | **5** | TX205,TX6252,TX775,TX777,TX779 | 17 |
| Other | **7** | H95,MO17,OH43,OH7B,S8324,Q381,NK787 | 41 |
| **South** | | | |
| CG | **5** | C103,CG102,CGR03,CG108,CGR01 | 7 |
| DK3IIH6 | 1 | DK3IIH6 | 23 |
| LH | **4** | LH162,LH51,LH210,LH38 | 25 |
| LH82 | 1 | LH82 | 76 |
| LH198 | 1 | LH198 | 10 |
| LH185 | 1 | LH185 | 32 |
| LH195 | 1 | LH195 | 695 |
| PH | **18** | PHN82,PHG35,PHM49,PHJ65,PHM57,PHN11,PHN47,PHR03,PHR55, PHR63,PHW30,PHW53,PHHV4,PB80,PHR25,PHG47,PHAJ0,PH207 | 86 |
| PHB | 1 | PHB47 | 56 |
| PHK | **1** | PHK76 | 4 |
| PHP | **1** | PHP60 | 5 |
| PHT | 2 | PHT69,PHTD5 | 322 |
| PHZ | 1 | PHZ51 | 522 |
| TX | **6** | TX205,TX6252,TX775,TX777,TX779,TX714 | 19 |
| Other | **5** | H95,MO17,OH43,NK787,Q381 | 25 |

* Number of unique hybrids produced by the testers

**Supplementary Table 2.** **Description of the environmental covariates simulated by APSIM.**

| **Num.** | **Variable** | **Units** | **Description** |
| --- | --- | --- | --- |
| 1 | TT | °C | Thermal Time |
| 2 | CumTT | °C | Accumulated thermal time since one day before germination |
| 3 | Eo | mm | Potential evapotranspiration of the whole soil-plant system |
| 4 | Eos | mm | Potential evaporation from soil surface |
| 5 | Es | mm | Actual (realized) soil water evaporation |
| 6 | ESW | mm | Extractable soil water from each layer relative to lower limit at 15Bar (LL15) |
| 7 | Flow | mm | Unsaturated water movement between layers |
| 8 | FlowNO_3_ | kg/ha | Amount of Nitrogen leaching as NO_3_ from each soil layer |
| 9 | Flux | mm | Saturated water flux from each layer to the layer below |
| 10 | Infiltration | mm | Daily infiltration across the soil surface |
| 11 | PotInf | mm | Potential infiltration, this is set by microclimate and is rainfall less that  intercepted by the canopy and residue component |
| 12 | PotRunoff | mm | Gets potential runoff |
| 13 | Runoff | mm | Daily runoff |
| 14 | SW | mm/mm | Amount of water in the soil in each layer |
| 15 | T | days | Time since start of second state evaporation |
| 16 | WaterTable | mm | Depth of the water table (10000 if no water table present) |
| 17 | CoverGreen | 0-1 | Total plant green cover from all organs |
| 18 | CoverTotal | 0-1 | Total plant cover from all organs |
| 19 | LAI | m2/m2 | Leaf area index, live plant green |
| 20 | Biomass | kg/ha | Above ground biomass |
| 21 | Yield | kg/ha | Grain yield dry weight |

Water variables ESW, Flow, FlowNO_3_, Flux, and SW are provided by APSIM by each layer. They are aggregated across all layers to get a total value.

**Supplementary Table 3.** **Phenological stages simulated by APSIM.**

| **Period** | **Start** | **End** | **Length (days)*** | | |
| --- | --- | --- | --- | --- | --- |
|  |  |  | **Mean** | **Min** | **Max** |
| GerEme | Germination | Emergence | 7.72 | 5 | 14 |
| EmeEnJ | Emergence | EndJuvenile | 18.6 | 11 | 38 |
| EnJFlo | EndJuvenille | FloralInitiation | 1.18 | 1 | 2 |
| FloFla | FloralInitiation | FlagLeaf | 38.91 | 32 | 52 |
| FlaFlw | FlagLeaf | Flowering | 4.67 | 4 | 6 |
| FlwStG | Flowering | StartGrainFill | 8.63 | 6 | 12 |
| StGEnG | StartGrainFill | EndGrainFill | 34.31 | 24 | 73 |
| EnGMat | EndGrainFill | Maturity | 1.76 | 1 | 5 |
| MatHar | Maturity | HarvestRipe | 1.68 | 1 | 4 |

*Mean, minimum and maximum length of the periods were calculated across all 136 year-locations (2014-2021)

**Supplementary Table 4**. **Analysis of variance for grain yield and flowering traits in southern trials using two models.**

|  | **Grain yield** | | **Days-to-anthesis** | | **Days-to-silking** | | **ASI** | |
| --- | --- | --- | --- | --- | --- | --- | --- | --- |
| **Source** | **Random effects** | **SNP+**  **EC** | **Random effects** | **SNP+**  **EC** | **Random effects** | **SNP+**  **EC** | **Random effects** | **SNP+**  **EC** |
| YEAR (Y) | 0.172 (0.116) | -- | 0.187 (0.078) | -- | 0.226 (0.131) | -- | 0.080 (0.044) | -- |
| LOC (L) | 0.199 (0.088) | -- | 0.273 (0.106) | -- | 0.290 (0.099) | -- | 0.091 (0.039) | -- |
| YL | 0.404 (0.110) | -- | 0.546 (0.109) | -- | 0.437 (0.064) | -- | 0.327 (0.053) | -- |
| (Total YL) | 0.574 (0.007) | -- | 0.853 (0.004) | -- | 0.842 (0.005) | -- | 0.350 (0.007) | -- |
| EC | -- | 0.644 (0.020) | -- | 0.844 (0.014) | -- | 0.858 (0.015) | -- | 0.343 (0.016) |
| Hybrid (G) | 0.064 (0.003) | -- | 0.064 (0.001) | -- | 0.063 (0.002) | -- | 0.034 (0.003) | -- |
| SNP | -- | 0.044 (0.007) | -- | 0.054 (0.007) | -- | 0.059 (0.007) | -- | 0.039 (0.006) |
| GxL | 0.038 (0.003) | -- | 0.012 (0.001) | -- | 0.013 (0.001) | -- | 0.056 (0.006) | -- |
| SNPxEC | -- | 0.096 (0.009) | -- | 0.038 (0.004) | -- | 0.039 (0.004) | -- | 0.080 (0.009) |
| Error | 0.309 (0.004) | 0.301 (0.003) | 0.067 (0.001) | 0.062 (0.001) | 0.079 (0.001) | 0.072 (0.001) | 0.561 (0.007) | 0.568 (0.006) |

Y: Year, L: Location, YL: Year-location, G: Hybrid, EC: Environmental Covariates. All traits were standardized to a unit variance. In parenthesis, the posterior standard deviation

**Supplementary Table 5**. **Phenotypic and genetic correlation (standard deviation) among traits in northern trials.**

|  |  | **Phenotypic Correlations** | | |  | **Correlation Estimates from  SNP+EC model** | | |
| --- | --- | --- | --- | --- | --- | --- | --- | --- |
| **Trait1** | **Trait2** | **Within**  **YL** | **Between**  **YL** | **Across**  **YL** |  | **Genetic** | **Environmental** | |
|  |  |  |  |  |  |  | **Within YL (Error)** | **Between YL (EC)** |
| Grain yield | Anthesis | -0.058  (0.004) | -0.108  (0.011) | -0.078  (0.004) |  | -0.031  (0.016) | -0.096  (0.004) | -0.100  (0.007) |
| Grain yield | Silking | -0.091  (0.004) | -0.128  (0.011) | -0.098  (0.004) |  | -0.055  (0.016) | -0.128  (0.004) | -0.121  (0.007) |
| Grain yield | ASI | -0.086  (0.004) | -0.159  (0.011) | -0.103  (0.004) |  | -0.160  (0.019) | -0.067  (0.004) | -0.181  (0.009) |
| Anthesis | Silking | 0.945  (0.001) | 0.992  (0.001) | 0.978  (0.001) |  | 0.988  (0.001) | 0.853  (0.003) | 0.993  (0.001) |
| Anthesis | ASI | -0.108  (0.004) | -0.003  (0.011) | -0.026  (0.004) |  | 0.337  (0.017) | -0.212  (0.004) | 0.022  (0.010) |
| Silking | ASI | 0.352  (0.004) | 0.121  (0.011) | 0.181  (0.004) |  | 0.476  (0.015) | 0.329  (0.004) | 0.144  (0.010) |

YL: Year-location. SNP+EC model includes SNPs plus environmental covariates (EC)

**Supplementary Table 6**. **Phenotypic and genetic correlation (standard deviation) among traits in southern trials.**

|  |  | **Phenotypic correlations** | | |  | **Correlation Estimates from  SNP+EC model** | | |
| --- | --- | --- | --- | --- | --- | --- | --- | --- |
| **Trait1** | **Trait2** | **Within**  **YL** | **Between**  **YL** | **Across**  **YL** |  | **Genetic** | **Environmental** | |
|  |  |  |  |  |  |  | **Within YL (Error)** | **Between YL (EC)** |
| Grain yield | Anthesis | 0.130 (0.007) | 0.223 (0.025) | 0.172 (0.007) |  | 0.491 (0.022) | -0.030 (0.007) | 0.235  (0.008) |
| Grain yield | Silking | 0.105 (0.007) | 0.210 (0.025) | 0.156 (0.007) |  | 0.468 (0.023) | -0.050 (0.007) | 0.225  (0.008) |
| Grain yield | ASI | -0.048 (0.007) | -0.077 (0.027) | -0.061 (0.007) |  | -0.015 (0.035) | -0.037 (0.007) | -0.053  (0.012) |
| Anthesis | Silking | 0.962 (0.002) | 0.990 (0.004) | 0.976 (0.002) |  | 0.986 (0.001) | 0.827 (0.004) | 0.991  (0.001) |
| Anthesis | ASI | -0.133 (0.007) | -0.018 (0.027) | -0.041 (0.007) |  | 0.175 (0.036) | -0.168 (0.007) | 0.018  (0.013) |
| Silking | ASI | 0.405 (0.006) | 0.122 (0.027) | 0.176 (0.007) |  | 0.335 (0.034) | 0.415 (0.006) | 0.154  (0.013) |

YL: Year-location. SNP+EC model includes SNPs plus environmental covariates (EC)

**Supplementary reference**

1. Becker, R. A., Wilks, A. R., Brownrigg, R., Minka, T. P. & Deckmyn, A. maps: Draw Geographical Maps. ﻿R package version 3.4.0 (2021). at <<https://CRAN.R-project.org/package=maps>>
