# Supplementary material for: Leveraging data from the Genomes-to-Fields Initiative to investigate genotype-by-environment interactions in maize in North America
Source: Nat Commun. 2023 Oct 30;14:6904. doi: 10.1038/s41467-023-42687-4 (PMC10616096; doi:10.1038/s41467-023-42687-4)
Supplement: Supplementary file 3 — Reporting Summary [file 41467_2023_42687_MOESM3_ESM.pdf]

nature portfolio

Corresponding author(s): Corresp: Gustavo de los Campos. Manuscript #NCOMMS-23-19987B

Last updated by author(s): Oct-06-2023

Reporting Summary

Nature Portfolio wishes to improve the reproducibility of the work that we publish. This form provides structure for consistency and transparency in reporting. For further information on Nature Portfolio policies, see our Editorial Policies and the Editorial Policy Checklist.

Statistics

For all statistical analyses, confirm that the following items are present in the figure legend, table legend, main text, or Methods section.

n/a	Confirmed	
☐	☒	The exact sample size (*n*) for each experimental group/condition, given as a discrete number and unit of measurement
☐	☒	A statement on whether measurements were taken from distinct samples or whether the same sample was measured repeatedly
☐	☒	The statistical test(s) used AND whether they are one- or two-sided<br>*Only common tests should be described solely by name; describe more complex techniques in the Methods section.*
☐	☒	A description of all covariates tested
☐	☒	A description of any assumptions or corrections, such as tests of normality and adjustment for multiple comparisons
☐	☒	A full description of the statistical parameters including central tendency (e.g. means) or other basic estimates (e.g. regression coefficient) AND variation (e.g. standard deviation) or associated estimates of uncertainty (e.g. confidence intervals)
☐	☒	For null hypothesis testing, the test statistic (e.g. *F*, *t*, *r*) with confidence intervals, effect sizes, degrees of freedom and *P* value noted<br>*Give P values as exact values whenever suitable.*
☐	☒	For Bayesian analysis, information on the choice of priors and Markov chain Monte Carlo settings
☒	☐	For hierarchical and complex designs, identification of the appropriate level for tests and full reporting of outcomes
☐	☒	Estimates of effect sizes (e.g. Cohen's *d*, Pearson's *r*), indicating how they were calculated

Our web collection on statistics for biologists contains articles on many of the points above.

Software and code

Policy information about availability of computer code

Data collection	Environmental covariates were derived using the Agricultural Production Systems sIMulator (APSIM Next Gen 2021.11.3.6921) crop model with the 'APSIMx' R-package (v2.3.1).
Data analysis	We used the aov() function of R's 'base' package (v4.3.1) for the analysis of variance for each environmental covariate. Mixed-effects models were fitted using the 'lme4' R-package (v1.1.34) . Likelihood ratio test was performed with the anova() function from the 'base' R-package (v4.3.1) and with the lrtest() function from the 'lmtest' R-package (v0.9.40). Genomic prediction models were fitted with the 'BGLR' R-package (v1.1.0). The number of independent tests was obtained using the 'poolr' R-package (v1.1.1). The PC analysis was performed using the eigen() function of the 'base' R-package (v4.3.1)

For manuscripts utilizing custom algorithms or software that are central to the research but not yet described in published literature, software must be made available to editors and reviewers. We strongly encourage code deposition in a community repository (e.g. GitHub). See the Nature Portfolio guidelines for submitting code & software for further information.

Data

Policy information about availability of data

All manuscripts must include a data availability statement. This statement should provide the following information, where applicable:

- Accession codes, unique identifiers, or web links for publicly available datasets
- A description of any restrictions on data availability
- For clinical datasets or third party data, please ensure that the statement adheres to our policy

All the phenotypic, agronomic, and metadata considered in this study are released every year and are available under Public Domain Dedication in the G2F website (https://www.genomes2fields.org/resources). Genotypic data was obtained from CyVerse Data Store (https://doi.org/10.25739/tq5e-ak26). Soil data was sourced from the Soil Survey Geographic Database (SSURGO, https://sdmdataaccess.nrcs.usda.gov). Weather data was downloaded from the NASA Langley Research Center (LaRC) POWER Project (https://power.larc.nasa.gov) funded through the NASA Earth Science/Applied Science Program. The aggregated curated data set (including the SNP genotypes, phenotypes, and ECs) is available at the Figshare repository (https://doi.org/10.6084/m9.figshare.22776806). The workflow used to curate the G2F data and to simulate the ECs is available through the GitHub repository at https://github.com/QuantGen/MAIZE-HUB

Research involving human participants, their data, or biological material

Policy information about studies with human participants or human data. See also policy information about sex, gender (identity/presentation), and sexual orientation and race, ethnicity and racism.

Reporting on sex and gender	N/A
Reporting on race, ethnicity, or other socially relevant groupings	N/A
Population characteristics	N/A
Recruitment	N/A
Ethics oversight	N/A

Note that full information on the approval of the study protocol must also be provided in the manuscript.

Field-specific reporting

Please select the one below that is the best fit for your research. If you are not sure, read the appropriate sections before making your selection.

☒ Life sciences ☐ Behavioural & social sciences ☐ Ecological, evolutionary & environmental sciences

For a reference copy of the document with all sections, see nature.com/documents/nr-reporting-summary-flat.pdf

Life sciences study design

All studies must disclose on these points even when the disclosure is negative.

Sample size	Phenotype, genotype, and metadata were downloaded from the G2F Maize GxE project public repositories (https://www.genomes2fields.org). These data sets consist of raw and unfiltered information from experiments conducted every year from 2014 to 2021 in many locations in the United States and include over 4,000 maize hybrids.
Data exclusions	We performed quality control filtering to the downloaded data which includes the removal of phenotypic records with at least one missing data in any of planting, harvesting, anthesis, or silking dates. We also removed yield outliers within year-location, defined as yield records greater than the 75th percentile plus 2.5 times the inter-quartile range or smaller than the 25th percentile minus 2.5 times the inter-quartile range. Likewise, we removed records with ASI greater than 15 days. The quality controlled data includes 78,686 records of 4,372 hybrids, tested over 8 years (from 2014 to 2021) and 38 locations in the United States.
Replication	The benchmark provided includes two internal cross validation schemes. One of them is a 'years assigned to folds' approach which is completely reproducible since years (folds) are fixed.
Randomization	A second cross validation used in this study assigns hybrids to folds. This procedure was performed completely at random. This fold assignment is provided along with the data for reproducibility
Blinding	Blinding was not applied in our study for data collection. The cross validation schemes were merely based on the investigator's choice

Reporting for specific materials, systems and methods

We require information from authors about some types of materials, experimental systems and methods used in many studies. Here, indicate whether each material, system or method listed is relevant to your study. If you are not sure if a list item applies to your research, read the appropriate section before selecting a response.

Materials & experimental systems

n/a	Involved in the study
☒	☐ Antibodies
☒	☐ Eukaryotic cell lines
☒	☐ Palaeontology and archaeology
☒	☐ Animals and other organisms
☒	☐ Clinical data
☒	☐ Dual use research of concern
☒	☐ Plants

Methods

n/a	Involved in the study
☒	☐ ChIP-seq
☒	☐ Flow cytometry
☒	☐ MRI-based neuroimaging

Plants

Seed stocks	N/A
Novel plant genotypes	N/A
Authentication	N/A
